# Development and external validation of a preoperative nomogram for predicting pathological locally advanced disease of clinically localized upper urinary tract carcinoma

**DOI:** 10.1002/cam4.2988

**Published:** 2020-04-06

**Authors:** Takashi Yoshida, Takashi Kobayashi, Takayuki Kawaura, Makito Miyake, Katsuhiro Ito, Hiroshi Okuno, Takashi Murota, Noriyuki Makita, Mutsushi Kawakita, Gen Kawa, Tomoki Kitawaki, Kiyohide Fujimoto, Hideyasu Matsuyama, Hiroaki Shiina, Haruhito Azuma, Osamu Ogawa, Hidefumi Kinoshita, Tadashi Matsuda

**Affiliations:** ^1^ Department of Urology and Andrology Kansai Medical University Kori Hospital Osaka Japan; ^2^ Department of Urology and Andrology Kansai Medical University Osaka Japan; ^3^ Department of Urology Kyoto University Graduate School of Medicine Kyoto Japan; ^4^ Department of Mathematics Kansai Medical University Osaka Japan; ^5^ Department of Urology Nara Medical University Kashihara Japan; ^6^ Department of Urology National Hospital Organization Kyoto Medical Center Kyoto Japan; ^7^ Department of Urology and Andrology Kansai Medical University General Medical Center Osaka Japan; ^8^ Department of Urology Kobe City Medical Center General Hospital Kobe Japan; ^9^ Department of Urology Saiseikai Noe Hospital Osaka Japan; ^10^ Department of Urology Graduate School of Medicine Yamaguchi University Ube Japan; ^11^ Department of Urology Shimane University School of Medicine Izumo Japan; ^12^ Department of Urology Osaka Medical College Takatsuki Japan

**Keywords:** nomogram, prognosis, renal pelvis, surgical procedure, ureter, urothelial carcinoma

## Abstract

**Objective:**

To develop and validate a preoperative nomogram to predict pathological locally advanced disease (pLAD) of clinically localized upper urinary tract urothelial carcinoma (UTUC) treated with extirpative surgery.

**Methods:**

In total, 1101 patients with cN0M0 UTUC (development cohort, n = 604; validation cohort, n = 497) from 2 independent academic databases were retrospectively analyzed. pLAD was defined as pT3/4 and/or pN+. Multivariate logistic regression was used to develop a nomogram. The accuracy of the nomogram was evaluated with a receiver operating characteristic curve, calibration plot, and decision curve analysis.

**Results:**

The development and validation cohorts comprised 204 (33.8%) and 178 (35.8%) patients with pLAD, respectively. The multivariate analyses showed that the neutrophil‐to‐lymphocyte ratio (hazard ratio [HR], 2.27; *P* < .001), chronic kidney disease (HR, 1.56; *P* = .032), tumor location (HR, 1.60; *P* = .029), hydronephrosis (HR, 2.71; *P* < .001), and local invasion on imaging (HR, 8.59; *P* < .001) were independent predictive factors. After bootstrapping, a well‐calibrated nomogram achieved discriminative accuracy of 0.77 in the development cohort. The decision curve analysis demonstrated improved risk prediction against threshold probabilities (≥8%) of pLAD. These results were consistent in the validation cohort.

**Conclusion:**

Our novel nomogram allows for more highly accurate prediction of pLAD of UTUC. This nomogram integrates standard imaging and laboratory factors that help to identify patients who will benefit from preoperative chemotherapy, extended lymph node dissection, or both.

## Graphical abstract

We developed a preoperative nomogram for the prediction of pathological locally advanced disease of clinically localized upper urinary tract carcinoma using five readily available variables based on standard imaging and laboratory findings. The use of our model will improve clinical decision‐making with regard to candidates for neoadjuvant chemotherapy and/or extended lymphnode dissection, even in patients who are omitted from ureteroscopy or who have insignificant tumor on imaging, prior to extirpative surgery for upper urinary tract carcinoma.

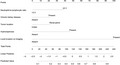

## 1 INTRODUCTION

Upper urinary tract urothelial carcinoma (UTUC) is a relatively uncommon disease, accounting for 5% to 10% of all urothelial carcinomas.^1^ Patients with advanced UTUC have a poor 5‐year cancer‐specific survival rate (<50% for pT2‐3 and <10% for pT4).^1^ Several postoperative nomograms are available to predict oncological outcomes in patients with UTUC, and these nomograms are thus useful for selecting patients who require adjuvant chemotherapy.^2^, ^3^, ^4^ To our knowledge, however, few preoperative models are available to predict pathological outcomes. The use of cisplatin‐based chemotherapy for high‐risk UTUC is preferable in a neoadjuvant setting because of the possible significant loss of renal function after radical nephroureterectomy (RNU), which may render a patient unfit for such chemotherapy.^5^ Further, extended lymph node dissection (LND) benefits patients with locally advanced UTUC.^6^ Thus, preoperative tools for predicting pathological outcomes are urgently required to identify patients with UTUC who are eligible for neoadjuvant chemotherapy (NAC), extended LND, or both.

Accurate preoperative staging of UTUC presents a clinical challenge. The current standard of staging is mainly based on imaging, which achieves limited accuracy. This limitation has led to the development of preoperative prediction models that incorporate pathological findings obtained by ureteroscopy (URS) to predict pathological locally advanced disease (pLAD).^7^, ^8^ In clinical practice, however, diagnostic URS is applied to a limited number of patients with UTUC (eg, patients with insignificant tumors on imaging or patients who are eligible for kidney‐sparing surgery), and it is currently performed for 32.1% to 44.8% of patients before RNU.^9^, ^10^, ^11^ Therefore, we believe that the development of an accurate preoperative pLAD prediction tool that is not completely dependent on URS findings will benefit more patients with UTUC. The aim of the present study was to develop and validate a preoperative nomogram that incorporates clinical predictors associated with pLAD in patients with cN0M0 UTUC because patients with grossly involved lymph nodes (cN+) are generally recommended to undergo NAC, extended LND, or both.

## 2 PATIENTS AND METHODS

### 2.1 Patient selection

An institutional review board approved this study, which was performed with all participating sites providing the necessary institutional data‐sharing agreements before the study commenced. The following two independent UTUC databases of patients who underwent RNU or segmental ureterectomy and who had histologically confirmed UTUC were analyzed in this study: the Kansai Medical University–Kyoto University Collaborative UTUC database (2004‐2016; development cohort, n = 1052) and the Nishinihon Uro‐Oncology Collaborative Group UTUC database^12^ (1994‐2015; validation cohort, n = 1438). We selected patients with cN0M0 UTUC who had not undergone preoperative chemotherapy or radiotherapy. The following variables of 1101 patients (development cohort, n = 604; validation cohort, n = 497) were analyzed: age, sex, Eastern Cooperative Oncology Group performance status, history of bladder cancer, neutrophil‐to‐lymphocyte ratio (NLR), estimated glomerular filtration rate (eGFR), hemoglobin concentration, local invasion on imaging, multifocality, tumor location, hydronephrosis, and pathological stage.

### 2.2 Variables

Blood parameters such as the neutrophil and lymphocyte counts, eGFR, and hemoglobin concentration were measured within 1 month before surgery. The NLR was calculated as the neutrophil count divided by the lymphocyte count.^13^ Chronic kidney disease (CKD) was defined as a preoperative eGFR of <60 mL/min/1.73 m^2^.^14^ Anemia was defined according to the World Health Organization classification as a hemoglobin concentration of ≤13 and ≤12 g/dL in men and women, respectively.^15^ Local invasion on imaging (ie, parenchymal, renal sinus fat, or periureteric invasion)^7^ (absent or present), multifocality (single or multiple), tumor location (renal pelvis or ureter), and hydronephrosis (absent or present) were confirmed using computed tomography or magnetic resonance imaging. Diagnostic URS findings were used only to detect multifocality and the tumor location if tumors were insignificant on imaging. If tumors were located in both the renal pelvis and ureter, the tumor location was defined according to the site of the largest tumor. At each institution, the clinical stage of the tumor was referenced according to the radiologists’ reports or URS findings (if tumors were judged insignificant), according to the 2002 TNM staging system of the American Joint Committee on Cancer/International Union against Cancer (AJCC/UICC).^16^

### 2.3 Surgical procedures and pathological evaluations

RNU was performed in accordance with the standard technique of extrafascial dissection of the kidney along the entire length of the ureter and adjacent segment of the bladder cuff. The hilar and regional lymph nodes adjacent to the ipsilateral great vessel were generally resected if intraoperatively palpable. The extent of lymphadenectomy was determined at the surgeon's discretion. Segmental ureterectomy was performed in imperative cases such as the presence of a solitary kidney, chronic renal failure, or impaired renal function, or in elective cases such as the presence of a low‐grade tumor restricted to the distal ureter (n = 24/1101). All surgical specimens were processed according to standard pathological procedures employed at each institution. The tumors were staged according to the 2002 AJCC/UICC TNM staging system,^16^ and the histological subtype was determined according to the World Health Organization criteria of 1973.^17^

### 2.4 Follow‐up protocol

Postoperative follow‐up comprised a routine physical examination, blood evaluation, computed tomography, cystoscopy, and cytological assessment. Patients were generally examined postoperatively every 3 months for 2 years, every 6 months for 2 years, and annually thereafter. Survival data were acquired from the patients’ medical records.

### 2.5 Statistical analysis

The endpoint of this study was pLAD (defined as pT3/4 and/or pN+ upon extirpative surgery). Continuous data are reported as median and range, and categorical data are reported as number and percentage of patients. The optimal cut‐off value of the NLR was chosen to maximize the sensitivity and specificity of predicting pLAD^18^ and was determined to be 2.3 (from the development cohort). Preoperative variables were compared between the development and validation cohorts using the chi‐square and Mann–Whitney tests, as appropriate. Backward step‐down selection was used to generate most of the informative nomograms with the fewest variables (ie, reduced models). The predictive accuracies of the models were analyzed using the area under the receiver operating characteristic curve (AUC). An internal validation was performed on 2000 samples using a bootstrapping technique to adjust for overfitting.^19^ Calibration plots were assessed by comparing the predicted probabilities with the actual observed frequencies. External validation was performed on the validation cohort to assess the discriminatory ability and calibrate the developed model. A decision curve analysis was performed to determine and compare the clinical net benefit among the prediction models.^20^ All statistical analyses were performed using the R open‐source statistical software package (www.r‐project.org/). A two‐sided *P* < 0.05 was considered statistically significant.

## 3 RESULTS

### 3.1 Patients’ characteristics

The clinicopathological characteristics of the development and validation cohorts are presented in Table 1. The cohorts exhibited significant differences in age, sex, Eastern Cooperative Oncology Group performance status, NLR, history of bladder cancer, anemia, and the population that underwent LND. The other variables were not significantly different between the two groups. Among the entire study population (n = 1101) 382 (34.7%) patients had pLAD. Among the patients with pLAD in the development cohort (n = 604), 187 (31.0%) had pT3, 16 (2.6%) had pT4, and 20 (3.3%) had pN+ disease. Among the patients with pLAD in the validation cohort (n = 497), 158 (31.8%) had pT3, 13 (2.6%) had pT4, and 22 (4.4%) had pN+ disease.

**TABLE 1:** Clinicopathological characteristics of 1101 patients with upper urinary tract carcinoma undergoing extirpative surgery

Variable	All patients	Development cohort	Validation cohort	*P* value
	(n = 1101)	(n = 604)	(n = 497)	
Age, years, median, (IQR)	73.0 (66.0‐79.0)	73.0 (67.0‐79.0)	72.0 (65.0‐78.0)	.037
Sex, n (%)				.034
Female	326 (29.6)	195 (32.3)	131 (26.4)	
Male	775 (70.4)	409 (67.7)	366 (73.6)	
ECOG PS, n (%)				<.001
0	933 (84.7)	438 (72.5)	482 (97.0)	
≥1	181 (16.4)	166 (27.5)	15 (3.0)	
History of bladder cancer, n (%)				.018
Absent	500 (74.4)	526 (87.1)	407 (81.9)	
Present	168 (15.3)	78 (12.9)	90 (18.1)	
Neutrophil to lymphocyte ratio, median, (IQR)	2.2 (1.9‐3.2)	2.3 (1.8‐3.3)	2.1 (1.6‐3.0)	.001
≤2.3, n (%)	576 (52.3)	293 (48.5)	283 (56.9)	.006
>2.3, n (%)	525 (47.7)	311 (51.5)	214 (43.1)	
Estimate glomerular filtration rate, ml/min/1.73 m^2^, median, (IQR)	58.0 (45.2‐71.5)	57.0 (44.8‐70.2)	59.1 (46.0‐72.9)	.129
Chronic kidney disease, n (%)				.203
Absent	515 (46.8)	272 (45.0)	243 (48.9)	
Present	586 (53.2)	332 (55.0)	254 (51.1)	
Hemoglobin, median, (IQR)	13.1 (11.7‐14.1)	12.9 (11.5‐14.0)	13.2 (11.9‐14.2)	.087
Anemia, n (%)				.036
Absent	717 (65.1)	410 (67.9)	307 (61.8)	
Present	384 (34.9)	194 (32.1)	190 (38.2)	
Local invasion on image, n (%)				.409
Absent	926 (84.1)	513 (84.9)	413 (83.1)	
Present	175 (15.9)	91 (15.1)	84 (16.9)	
Multifocality, n (%)				.096
Single	949 (86.2)	511 (84.6)	438 (88.1)	
Multiple	152 (13.8)	93 (15.4)	59 (11.9)	
Tumor location, n (%)				.762
Renal pelvis	564 (51.2)	312 (51.7)	252 (50.7)	
Ureter	537 (48.8)	292 (48.3)	245 (49.3)	
Hydronephrosis, n (%)				.333
Absent	547 (49.7)	292 (48.3)	255 (51.3)	
Present	554 (50.3)	312 (51.7)	242 (48.7)	
Lymph node dissection performed, n (%)	734 (66.7)	429 (71.0)	305 (61.4)	.001
Pathological locally advanced disease, n (%)^a^	382 (34.7)	204 (33.8)	178 (35.8)	.485
Recurrence, n (%)	248 (22.7)	132 (21.9)	116 (23.7)	.513
Cancer‐specific mortality, n (%)	156 (14.2)	85 (14.1)	71 (14.3)	.931

Abbreviations: ECOG PS, Eastern Cooperative Oncology Group performance status; IQR, interquartile range.

^a^ pT3/4 and/or pN+.

### 3.2 Univariate and multivariate models predicting pLAD

Table 2 shows the results of the univariate and multivariate logistic regression analyses used to predict the likelihood of pLAD upon extirpative surgery. The univariate analysis revealed that a history of bladder cancer, a high NLR, CKD, anemia, local invasion on imaging, and hydronephrosis were significant factors associated with pLAD. After implementation of a backward step‐down selection process in the multivariate analysis, the best reduced model was found to incorporate the NLR, CKD, local invasion on imaging, tumor location, and hydronephrosis.

**TABLE 2:** Univariate and multivariate logistic regression models predicting pathological locally advanced disease

Variable	Univariate		Multivariate	
	OR (95% CI)	*P* value	OR (95% CI)	*P* value
Age (continuous value)	1.00 (0.99‐1.02)	.877	–	–
Sex (male vs female)	1.07 (0.74‐1.53)	.732	–	–
ECOG PS (≥1 vs 0)	0.93 (0.63‐1.35)	.691	–	–
History of bladder cancer (present vs absent)	0.55 (0.31‐0.96)	.034	–	–
Neutrophil to lymphocyte ratio (≥2.3 vs <2.3)	2.49 (1.75‐3.54)	<.001	2.27 (1.54‐3.35)	<.001
Chronic kidney disease (present vs absent)	1.89 (1.34‐2.68)	<.001	1.56 (1.04‐2.23)	.032
Anemia (present vs absent)	1.47 (1.03‐2.09)	.035	–	–
Local invasion on imaging (present vs absent)	8.78 (5.23‐14.8)	<.001	8.59 (4.93‐15.0)	<.001
Multifocality (multiple vs solitary)	0.77 (0.48‐1.25)	.294	–	–
Tumor location (ureter vs pelvis)	1.33 (0.95‐1.87)	.098	1.60 (1.05‐2.43)	.029
Hydronephrosis (present vs absent)	2.46 (1.74‐3.50)	<.001	2.71 (1.76‐4.16)	<.001

Abbreviations: CI, confidence interval; ECOG PS, Eastern Cooperative Oncology Group performance status; OR, odds ratio.

### 3.3 Development and validation of a predictive nomogram

**FIGURE 1:**
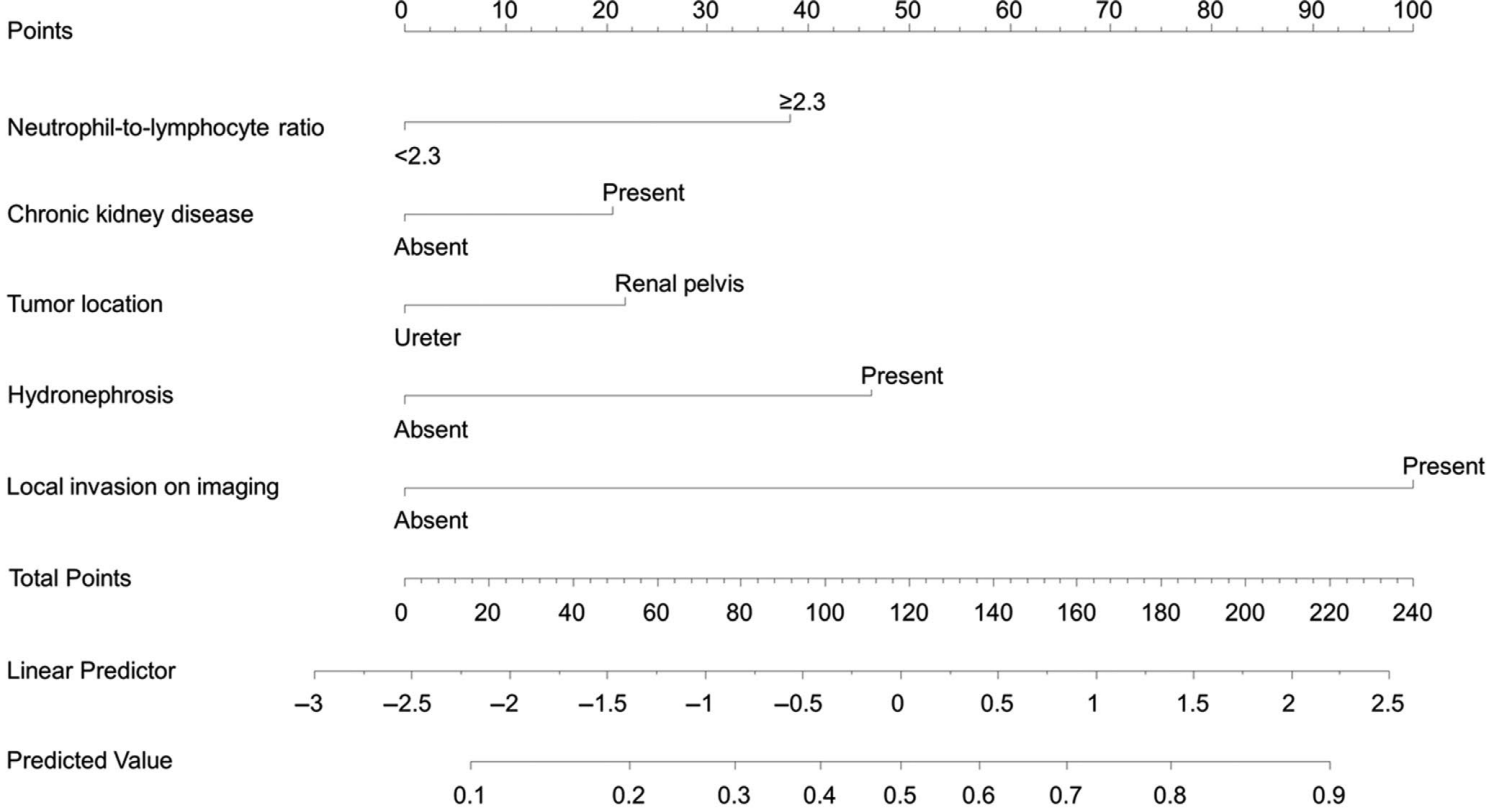
Nomogram using a reduced multivariate model for predicting pathological locally advanced disease upon extirpative surgery

**FIGURE 2:**
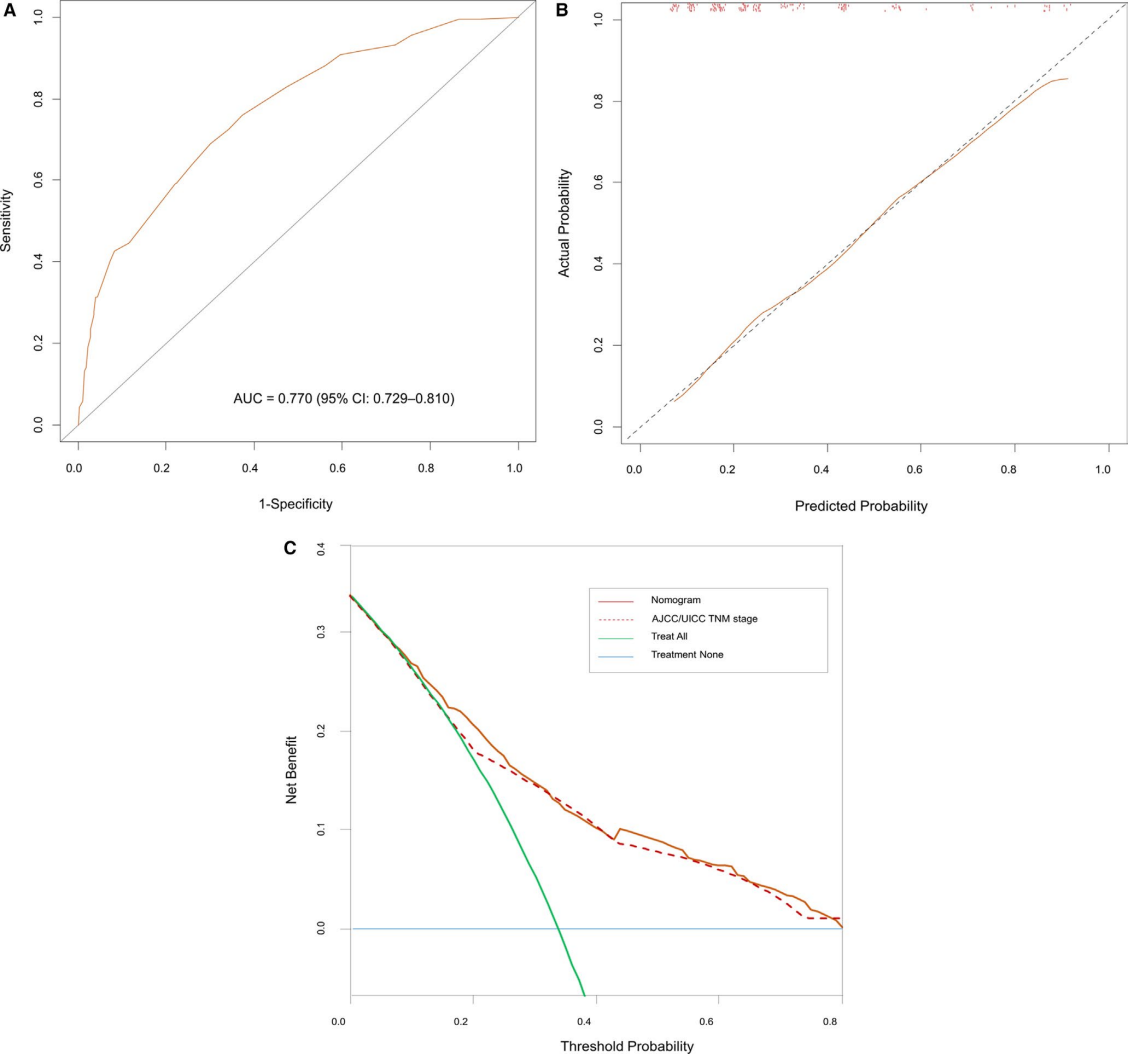
(A) Receiver operating characteristic curve analysis for evaluating the accuracy of the nomogram. (B) Calibration plot depicting the correlation between nomogram‐predicted probability and actual pathological locally advanced disease as internal validation. (C) Decision curve analysis demonstrating the net benefit of the nomogram compared with that of the American Joint Committee on Cancer/International Union against Cancer (AJCC/UICC) TNM staging system for predicting pathological locally advanced disease

The reduced model incorporating five readily available factors was used to generate a preoperative nomogram (Figure 1). After bootstrapping for the internal validation, the AUC of this model was 0.77 (95% confidence interval [CI], 0.73‐0.81) (Figure 2A), and the calibration plots revealed minimal differences between the nomogram‐predicted probabilities and the observed proportions (Figure 2B). In the decision curve analysis, the nomogram offered a net benefit over the “treat all” strategy at a threshold probability of ≥8% and well across a wide range of threshold probabilities compared with the conventional AJCC/UICC TNM staging (Figure 2C). When using a cut‐off of ≥45.0%, the nomogram predicted a high risk of pLAD with a 0.66 maximum percentage of correct classifications.

**FIGURE 3:**
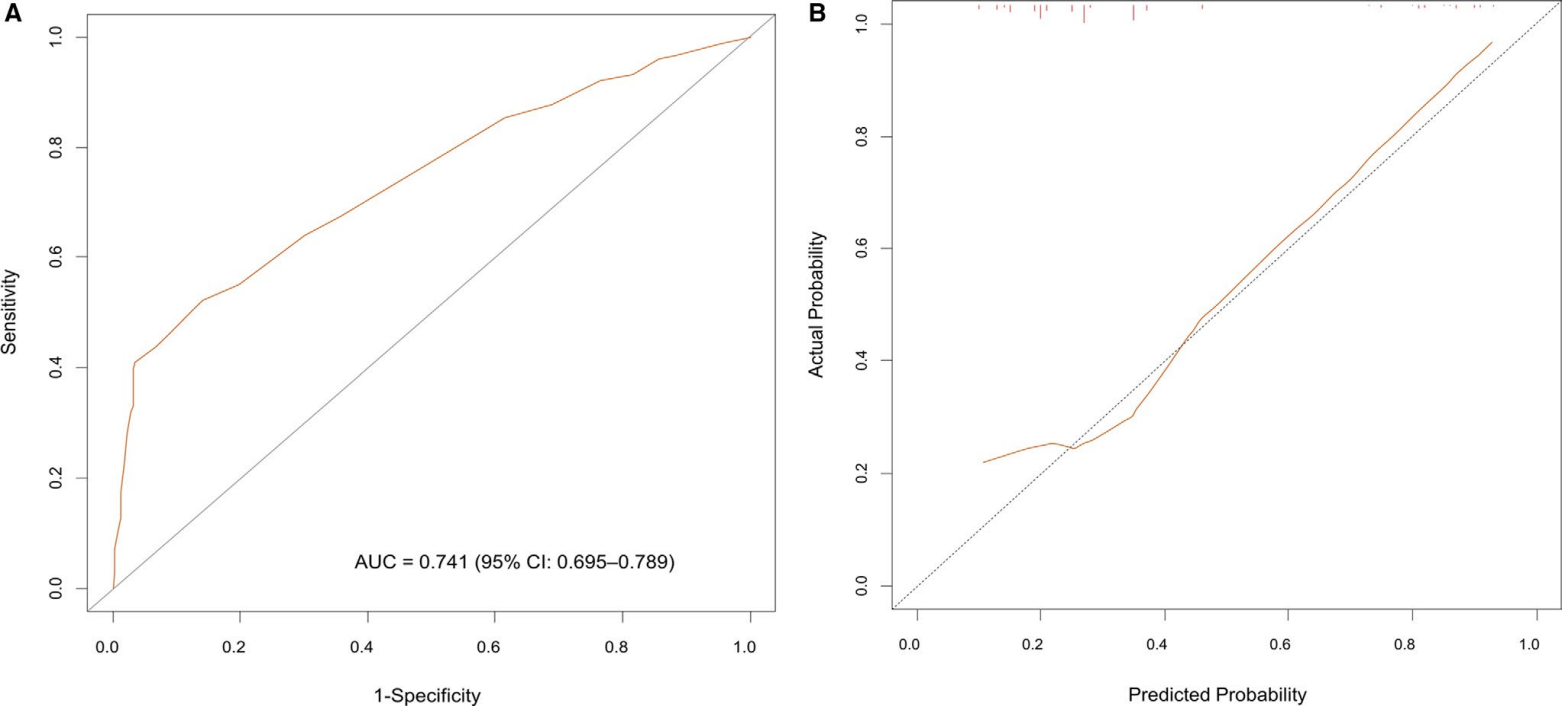
(A) Receiver operating characteristic curve analysis for evaluating the accuracy of the nomogram. (B) Calibration plot depicting the correlation between nomogram‐predicted probability and actual pathological locally advanced disease as external validation

In the external validation, the discriminative accuracy of the model was 0.74 (95% CI, 0.70‐0.79) (Figure 3A), and the calibration plots showed no significant deviation from the ideal prediction (Figure 3B).

### 3.4 Subgroup evaluation of the nomogram

Because local invasion on imaging is a well‐established clinical factor for estimating pLAD, we additionally tested the predictive ability of this nomogram when applied to patients with nonlocal invasion on imaging (development cohort, n = 513; validation cohort, n = 413). The discrimination of this nomogram was 0.70 (95% CI, 0.65‐0.75), and the calibration plots showed no significant deviation from the ideal prediction in the development cohort (Figure S1A In the external validation cohort, the nomogram showed an accuracy of 0.60 (95% CI, 0.54‐0.66), and the calibration plots deviated <10% from the ideal prediction (Figure S1B).

## 4 DISCUSSION

In this study, we developed a novel nomogram for predicting pLAD before surgical treatment of cN0M0 UTUC. This model was well calibrated and showed minimal deviations between the predicted and actual outcomes in the validation cohort. Further, the model estimated pLAD in patients with nonlocal invasion on imaging. The use of our model will likely improve clinical decision‐making to select candidates for NAC and/or extended LND, even patients in whom URS is omitted or who have an insignificant tumor on imaging before extirpative surgery for UTUC.

The strengths of our novel nomogram include the use of a large development cohort comprising complete data for cN0M0 UTUC and an independent validation cohort of equivalent size. Excellent reproducibility of the internal validation using the bootstrapping method and external validation indicates high reliability of our model. Further, our nomogram achieved a higher net benefit than that of the AJCC/UICC TNM staging system routinely used in current practice, indicating that the nomogram is clinically relevant and may change clinical decisions in certain cases. Moreover, our model does not include URS findings, making it widely applicable and generalizable to patients with UTUC detected by imaging upon the initial screening. Nonetheless, the accuracy of our nomogram (0.77) was comparable with that of two previous models using URS findings.^7^, ^8^

We often encounter discrepancies between the clinical and pathological stages of UTUC because of the lack of specific biomarkers or imaging systems. Computed tomography and magnetic resonance imaging are mainly used for clinical staging according to the AJCC/UICC TNM staging system, particularly to differentiate between organ‐confined tumors and locally advanced tumors and to detect lymph node enlargement.^1^, ^21^ Nevertheless, it is difficult to detect flat tumors and microscopic invasion and to distinguish unclear images associated with inflammatory changes around tumors, leading to under‐ or over‐staging.^21^ Incorporation of other clinically relevant factors into the prediction model for pLAD is therefore reasonable to compensate for such limitations of imaging.

In this study, hydronephrosis and the tumor location were independently associated with pLAD. These findings are consistent with those of a previous study showing that the presence of hydronephrosis predicts an aggressive tumor phenotype.^22^ However, other studies have produced conflicting results. For example, two studies^8^, ^23^ showed that patients with renal pelvic tumors are at higher risk of pLAD than patients with ureteral tumors, as did our study, whereas another study showed the opposite tendency.^24^ The reason for this this discrepancy is unclear. We hypothesize that renal pelvic tumors tend to progress asymptomatically to an advanced stage, whereas ureteral carcinomas tend to be diagnosed earlier due because of associated symptoms such as hydronephrosis.^8^, ^23^

To the best of our knowledge, this is the first study to generate a preoperative nomogram for prediction of UTUC using standard laboratory variables such as the NLR and eGFR (considered CKD). Numerous studies have shown that the preoperative NLR, which may reflect tumor‐associated inflammation and host immunity, is significantly associated with UTUC progression in terms of lymphovascular invasion, tumor T stage, and tumor grade.^13^ Moreover, the NLR played an important role in improving the predictive ability of our model. Preoperative CKD is an important factor for predicting pLAD^14^ as well as an indicator of ineligibility for adjuvant cisplatin‐based chemotherapy regimens because of eGFR loss after RNU (median, 32%).^25^ Therefore, for patients with preoperative CKD, the prediction of pLAD is mandatory to determine whether preoperative cisplatin‐based chemotherapy is required to improve their oncological outcomes.

URS, which is a useful tool for diagnosing and confirming the tumor grade of UTUC, is strongly recommended for patients with undetectable or unclear tumors on imaging and patients who are considered eligible for kidney‐sparing surgery.^1^ Two preoperative predictive models that incorporate URS findings are available for predicting pLAD according to current guidelines.^1^ The model established by Favaretto et al^7^ incorporates local invasion on imaging and high‐grade URS to identify nonorgan‐confined UTUC (AUC = 0.70). The model established by Margulis et al,^8^ which combines the URS findings of location, grade, and tumor architecture to predict pLAD, achieved an accuracy of 0.77.

However, the URS biopsy procedure has the following disadvantages: the risk of obtaining insufficient biopsy material for accurate evaluation, which occurs in ~40% of cases^26^; the potential need to upgrade clinical low‐grade cases on biopsy at the time of radical surgery, which occurs in 51% of cases^27^; high omission rates of ~37.2% to 67.9% in the real‐world clinical setting^9^, ^10^, ^11^; and concerns about delaying radical surgery.^28^, ^29^ Thus, it does not seem practical to perform preoperative URS in all patients with UTUC. In contrast, the parameters used in the present model are readily available during routine preoperative evaluation. Thus, it is reasonable to use models that incorporate URS findings for preoperative prediction of pLAD as well as our model, particularly when URS is required for the above‐mentioned reasons, including tumor documentation or pathological confirmation.

This study has several limitations, including its retrospective design and use of multi‐institutional databases. Additionally, the surgeries were performed by multiple surgeons at each institution with or without LND, and the extent of LND was selected at the physician's discretion. Centralized pathological and radiological reviews were not performed. We elected to exclude urinary cytology from the predictive model, largely because these data were only available for 53% of patients in our development cohort. Because of the lack of detailed clinical TNM staging data in the validation cohort, comparison of the net benefit between the nomogram and TNM staging could not be performed as an external validation. Undoubtedly, further improvements of our predictive model will be required by incorporating advanced imaging modalities and novel molecular biomarkers.

## 5 CONCLUSIONS

We established a preoperative nomogram to predict pLAD of UTUC using five readily available variables acquired from standard imaging and laboratory data. Our nomogram can facilitate collaborative clinical decision‐making (ie, selecting candidates for NAC, extended LND, or both) before extirpative surgery and may therefore improve the oncological outcomes in patients with localized UTUC.

## CONFLICT OF INTERESTS

The authors declare that they have no competing interests.

## AUTHOR CONTRIBUTIONS

Tadashi Matsuda had full access to all the data in the study and takes responsibility for the integrity of the data and the accuracy of the data analysis. Study concept and design: Yoshida, Kobayashi. Acquisition of data: All authors. Analysis and interpretation of data: Yoshida, Kobayashi, Kawaura. Drafting of the manuscript: All authors. Critical revision of the manuscript for important intellectual content: All authors. Statistical analysis: Kawaura, Kitawaki, Miyake, Obtaining funding: None. Administrative, technical, or material support: None. Supervision: Matsuda.

## Supporting information

Fig S1

Fig S2

## Data Availability

The data that support the findings of this study are available on request from the corresponding author. The data are not publicly available because of privacy or ethical restrictions.
