# Supplementary figures and images for: Development and external validation of a preoperative nomogram for predicting pathological locally advanced disease of clinically localized upper urinary tract carcinoma
Source: Cancer Med. 2020 Apr 6;9(11):3733–41. doi: 10.1002/cam4.2988 (PMC7286474; doi:10.1002/cam4.2988)

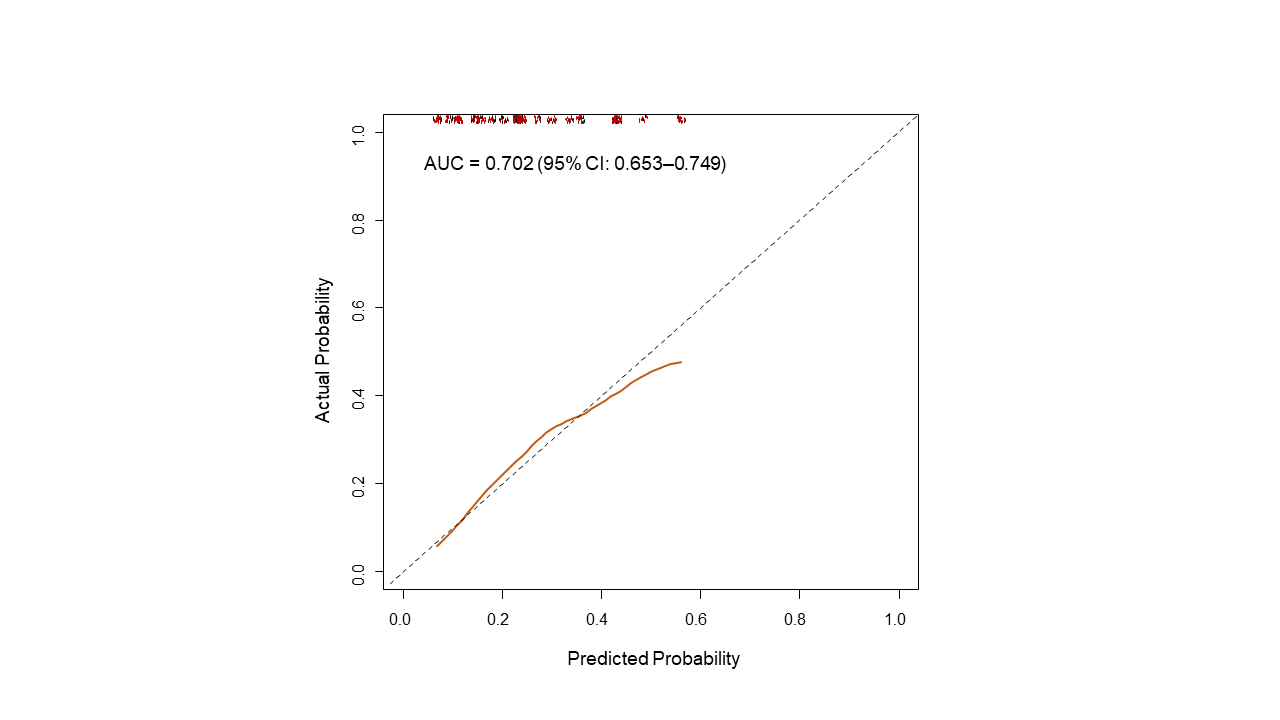

Supplement: Supplementary file 1 — Fig S1 [file CAM4-9-3733-s001.tif]

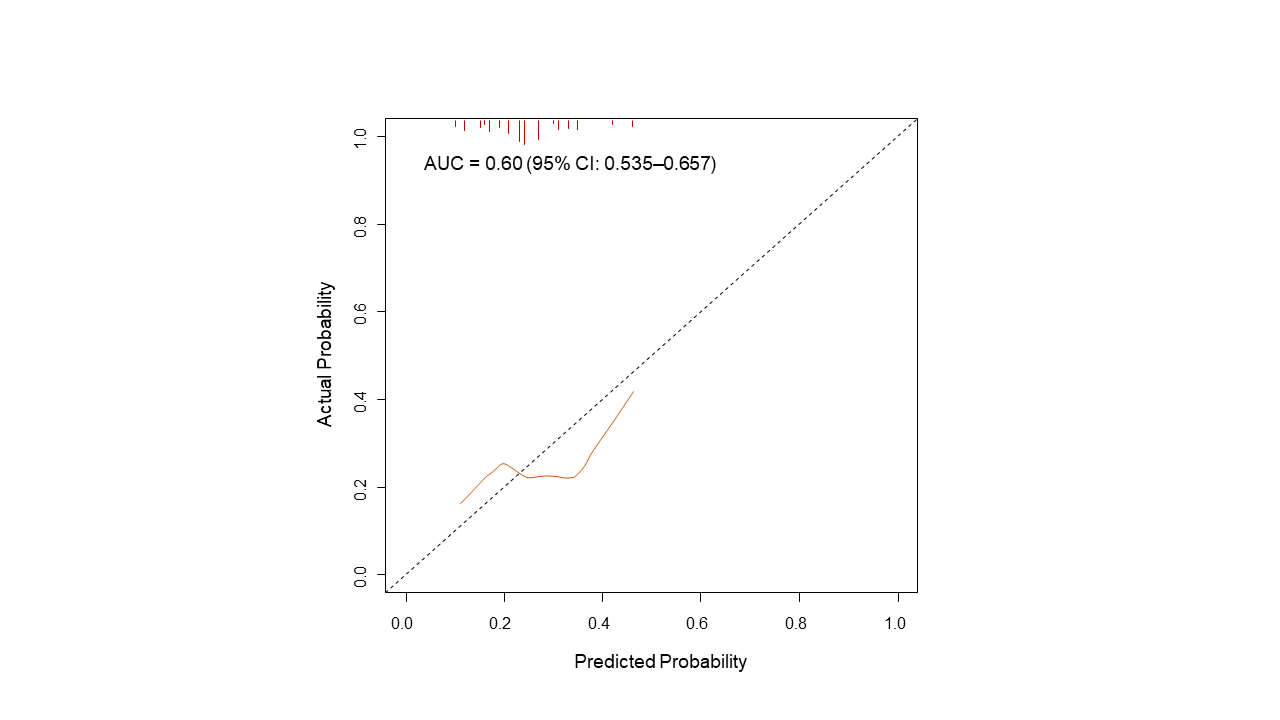

Supplement: Supplementary file 2 — Fig S2 [file CAM4-9-3733-s002.tif]
